# Chargenprüfung als wesentliche Säule der Versorgung mit sicheren und wirksamen Impfstoffen

**DOI:** 10.1007/s00103-022-03611-1

**Published:** 2022-10-20

**Authors:** Hanna Sediri-Schön, Jeannette Lange, Elena Grabski, Ralf Wagner, Eberhard Hildt

**Affiliations:** 1 Abteilung Virologie, Paul-Ehrlich-Institut, Langen, Deutschland; 2 Bundesinstitut für Impfstoffe und biomedizinische Arzneimittel, Abteilung Virologie, Paul-Ehrlich-Institut, Paul-Ehrlich-Straße 51–59, 63225 Langen, Deutschland

**Keywords:** SARS-CoV2, Chargenfreigabe, OMCL, COVID-19-Impfstoffe, OCABR, SARS-CoV2, Batch release, OMCL, COVID-19 vaccines, OCABR

## Abstract

Das Paul-Ehrlich-Institut (PEI) spielt eine zentrale Rolle bei der Freigabe von Impfstoffen in Deutschland, aber auch in Europa. Die experimentelle Prüfung und Freigabe der Chargen erfolgt nach dem Verfahren und den Regelungen der Chargenfreigabe der offiziellen Kontrollbehörden (Official Control Authority Batch Release, OCABR) sowie dem § 32 des deutschen Arzneimittelgesetzes (AMG). Die unabhängige Prüfung zielt darauf ab, die Konformität der in der Zulassung festgelegten Qualitätskriterien für jede hergestellte Charge nachzuweisen. Dieser Beitrag beschreibt das Verfahren der Chargenfreigabe im Allgemeinen sowie speziell für die während der COVID-19-Pandemie neu entwickelten und zugelassenen COVID-19-Impfstoffe.

## Abstract (en)

The Paul-Ehrlich Institute (PEI) plays a central role in the release of vaccines in Germany as well as Europe. The experimental testing and release of each vaccine batch is carried out according to the procedures and regulations of the Official Control Authority Batch Release (OCABR) and the German medicine act paragraph 32. The independent testing aims to demonstrate the conformity of quality criteria set in the marketing authorization for each lot produced. This article illustrates both the batch release procedure in general and specifically for the newly developed and approved COVID-19 vaccines during the COVID-19 pandemic.

## Die COVID-19-Pandemie und der daraus resultierende Impfstoffbedarf

Im Dezember 2019 wurde in der Stadt Wuhan (China) der erste COVID-19-Fall, verursacht durch das Coronavirus SARS-CoV‑2, gemeldet. Weitere Infektionen folgten und die Weltgesundheitsorganisation (WHO) erklärte den Ausbruch im März 2020 offiziell zur Pandemie. Seit dem ersten beobachteten Fall wurden trotz globaler Eindämmungsmaßnahmen (wie etwas Lockdowns) weltweit mehr als 520 Mio. Menschen infiziert und mehr als 6 Mio. Todesfälle gemeldet [1, 2].

Diese rasante Ausbreitung der Pandemie und die hohe Zahl an Todesopfern machten die Entwicklung und Verteilung sicherer und wirksamer COVID-19-Impfstoffe dringend notwendig. Bis August 2022 haben folgende COVID-19-Impfstoffe eine bedingte europäische Zulassung erhalten: Comirnaty von BioNTech/Pfizer (21.12.2020), Spikevax (ehemals COVID-19 Vaccine Moderna) von Moderna (06.01.2021), Vaxzevria (ehemals COVID-19 Vaccine AstraZeneca) von AstraZeneca (29.01.2021), Jcovden (ehemals COVID-19 Vaccine Janssen) von Janssen-Cilag International (11.03.2021), Nuvaxovid von Novavax (20.12.2021) und COVID-19 Vaccine Valneva von Valneva (24.06.2022; [3]). Zugelassen seit Anfang September 2022 sind die an die zirkulierenden Virusvarianten angepassten bivalenten Impfstoffe Comirnaty Original/Omicron BA.1 (01.09.2022) und Comirnaty Original/Omicron BA.4–5 (12.09.2022) von BioNTech/Pfizer sowie Spikevax bivalent Original/Omicron BA.1 (01.09.2022) von Moderna (Tab. 1).

**Table 1:** 

Impfstoff	Zulassungsinhaber	Zulassungsdatum
*Comirnaty*	BioNTech Manufacturing GmbH	21.12.2020
*Comirnaty* *Original/Omicron BA.1*		01.09.2022
*Comirnaty* *Original/Omicron BA.4‑5*		12.09.2022
*Spikevax *(ehemals COVID-19 Vaccine Moderna)	Moderna Biotech Spain S. L.	06.01.2021
*Spikevax bivalent Original/Omicron BA.1*		01.09.2022
*Vaxzevria *(ehemals COVID-19 Vaccine AstraZeneca)	AstraZeneca AB	29.01.2021
*Jcovden *(ehemals COVID-19 Vaccine Janssen)	Janssen-Cilag International NV	11.03.2021
*Nuvaxovid*	Novavax CZ, a.s.	20.12.2021
*COVID-19 Vaccine Valneva*	Valneva Austria GmbH	24.06.2022

Um die Ausbreitung der Pandemie einzudämmen und eine Rückkehr des öffentlichen Lebens zur Normalität zu ermöglichen, haben viele Länder aktiv die Beschaffung von COVID-19-Impfstoffen sowie die Durchführung von Impfungen organisiert und vor dem Hintergrund nachlassender Immunität und des Auftretens neuer Virusvarianten auch die Möglichkeit von Auffrischungsimpfungen seit Ende 2021 angeboten.

Dabei ist zu berücksichtigen, dass die Zulassung nicht die einzige notwendige Voraussetzung ist, damit ein COVID-19-Impfstoff in Europa bzw. in Deutschland in den Verkehr gebracht werden darf. Impfstoffe unterliegen einer stetigen Kontrolle und einer ständigen Überwachung. Bevor eine produzierte Impfstoffcharge für den europäischen bzw. deutschen Markt freigegeben wird, muss eine Chargenprüfung durchgeführt werden.

### Chargenprüfungsverfahren für Humanimpfstoffe

#### Rechtliche Grundlage

Für die Bundesrepublik Deutschland ist laut § 32 des deutschen Arzneimittelgesetzes (AMG) festgelegt, dass die Chargen eines zugelassenen Impfstoffs, unbeschadet der Zulassung, nur in den Verkehr gebracht werden dürfen, wenn sie von der zuständigen Bundesoberbehörde freigegeben worden sind. Dafür muss eine staatliche Chargenprüfung ergeben haben, dass die Charge nach Herstellungs- und Kontrollmethoden, die dem jeweiligen Stand der wissenschaftlichen Erkenntnisse und der Zulassung entsprechen, hergestellt und geprüft worden ist und dadurch die erforderliche Qualität festgestellt werden konnte. Die zuständige Bundesoberbehörde hat innerhalb einer Frist von 2 Monaten nach Eingang der zu prüfenden Chargenprobe eine Entscheidung für die Freigabe zu treffen. Eine Charge ist auch dann freizugeben, wenn die zuständige Behörde eines anderen Mitgliedstaats der Europäischen Union (EU) nach einer experimentellen Untersuchung festgestellt hat, dass die oben genannten Voraussetzungen vorliegen [4].

Auf europäischer Ebene erfolgt die Chargenfreigabeprüfung auf Basis der spezifischen europäischen Direktive 2001/83/EG Artikel 114, geändert durch die Direktive 2004/27/EG. Dieser Artikel besagt, dass ein Labor eines Mitgliedstaats eine Charge eines immunologischen Arzneimittels vor dem Inverkehrbringen testen kann, aber nicht muss [5, 6].

**Figure 1:**
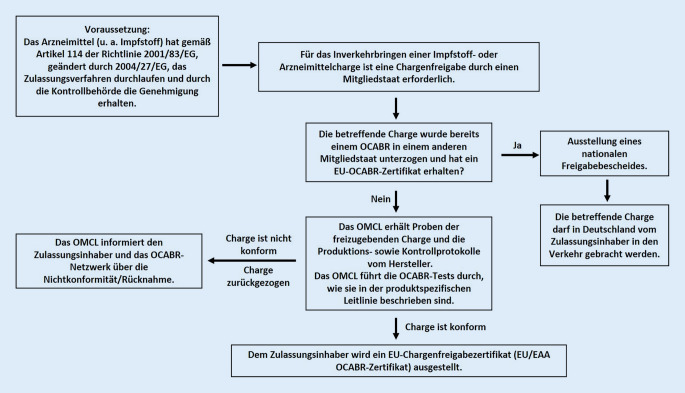

Ein Mitgliedstaat kann den Zulassungsinhaber vor dem Inverkehrbringen eines Impfstoffs dazu auffordern, Proben von jeder Charge für die Untersuchung durch ein amtliches Arzneimittelkontrolllabor (Official Medicines Control Laboratory, OMCL) einzureichen. Die zuständige Behörde des jeweiligen Mitgliedstaats stellt ein Chargenfreigabezertifikat (Batch Release Certificate) aus, wenn die Ergebnisse den Spezifikationen entsprechen. Dieses Verfahren ist bekannt als „Official Control Authority Batch Release“ (OCABR) und besteht aus analytischen Kontrollen und Dokumentenprüfungen, die für jede Impfstoffcharge zusätzlich zur herstellerspezifischen Chargenfreigabe und Kontrolle beim Hersteller durchgeführt werden müssen. Die Mitgliedstaaten stellen sicher, dass eine OCABR innerhalb von 60 Tagen nach Erhalt der Proben abgeschlossen ist. Weiterhin verlangen die Direktiven von den Mitgliedstaaten, die durch die offizielle Kontrollbehörde in einem anderen Mitgliedstaat durchgeführte OCABR anzuerkennen („mutual recognition“). Das heißt, im Falle einer Charge, die zuvor von einem anderen Arzneimittelkontrolllabor geprüft und deren Konformität mit den genehmigten Spezifikationen bestätigt wurde, wird die Chargenprüfung für diese bestimmte Charge nicht wiederholt. Sobald eine Charge von der zuständigen Behörde eines Mitgliedstaats freigegeben wurde, ist diese OCABR somit für alle anderen Mitgliedstaaten gültig und wird von allen anderen Mitgliedstaaten, die eine OCABR für dieses Produkt verlangen, anerkannt (Abb. 1).

Das EU-Chargenfreigabezertifikat (EU Official Control Authority Batch Release Certificate, EU/EEA-OCABR-Zertifikat) wird dem Zulassungsinhaber ausgestellt. Dieser muss sicherstellen, dass eine Kopie dieses Zertifikats den zuständigen Behörden der Mitgliedstaaten, in denen die Charge vermarktet werden soll, zur Verfügung gestellt wird. Neben der Kopie des OCABR-Zertifikates muss der Zulassungsinhaber auch das entsprechende MIF-Formular (Marketing Information Form) an die zuständige Behörde in dem/den Mitgliedstaat(en) senden, in dem/denen die Charge oder ein Teil der Charge des Impfstoffs in Verkehr gebracht werden soll. Nach Übersendung dieser Unterlagen und wenn die zuständige Behörde in diesem Mitgliedstaat innerhalb von 7 Arbeitstagen keine Einwände erhoben hat, kann die Charge dort vermarktet werden. Um eine Charge in Deutschland auf den Markt zu bringen, muss der Zulassungsinhaber jedoch zusätzlich einen nationalen Freigabebescheid beantragen (Abb. 1; [4–8]).

Eine Liste der OMCL in der EU, die derzeit die OCABR durchführen, ist auf der Internetseite des Europäischen Direktorats für die Qualität von Arzneimitteln und HealthCare (European Directorate for the Quality of Medicines and HealthCare, EDQM) erhältlich und wird regelmäßig aktualisiert [8].

#### Definition und Aufgaben eines OMCL

Die EU-Kommission und der Europarat haben am 26.05.1994 beschlossen, ein OMCL-Netzwerk einzurichten, welches zu einer neuen Zusammenarbeit im Bereich der Qualitätskontrolle von vermarkteten Human- und Tierarzneimitteln beitragen soll. 1995 übernahm das EDQM als technisches Sekretariat den Aufbau des OMCL-Netzwerkes [9].

Per Definition ist ein OMCL eine öffentliche Einrichtung, die im Auftrag der zuständigen Behörden und in Erfüllung anderer nationaler Verpflichtungen offizielle Laboruntersuchungen von Arzneimitteln (und verwandten Materialien wie Wirkstoffen, Hilfsstoffen, Plasmapools usw.) durchführt. Dies erfolgt unabhängig von den Herstellern oder Zulassungsinhabern. Die Prüfung der Produkte erfolgt im Interesse der behördlichen Marktüberwachung von Arzneimitteln in Bezug auf die Sicherheit von Mensch und/oder Tier vor und/oder nach dem Inverkehrbringen der jeweiligen Arzneimittel und ist nachweislich frei von Interessenkonflikten. Die Einbeziehung der OMCL in den Regulationsprozess ist ergänzend zur Bewertung des Zulassungsantrags und Teil der ständigen Überwachung der vermarkteten Produkte und soll sicherstellen, dass die für das Arzneimittel festgelegten Spezifikationen in der für die Vermarktung vorgesehenen Produktion erfüllt werden. Da diese offiziellen Laboruntersuchungen unabhängig von denjenigen durchgeführt werden, die an der Produktentwicklung, -herstellung und/oder -vermarktung beteiligt waren, werden zusätzliche wissenschaftliche Erkenntnisse zu einem Arzneimittel zum Nutzen des Patienten hinzugefügt. Eine unabhängige Analyse von Arzneimitteln, Wirkstoffen, Hilfsstoffen und anderen Materialien durch die OMCL soll sicherstellen, dass nur Arzneimittel auf den Markt kommen, die den in der Zulassung festgelegten Spezifikationen entsprechen. Damit tragen die OMCL wesentlich zum Schutz der Gesundheit von Mensch und Tier bei [10].

Das Paul-Ehrlich-Institut (PEI) ist als Bundesinstitut für Impfstoffe und biomedizinische Arzneimittel für die produktspezifische Prüfung dieser Arzneimittelproduktklassen zuständig und testet sie in seiner Rolle als OMCL nach festgelegten Methoden, um Qualität, Wirksamkeit und Unbedenklichkeit sicherzustellen. In Deutschland unterliegen nach § 32 AMG alle Impfstoffe der staatlichen Chargenprüfung durch die zuständige Bundesoberbehörde – dem PEI. Die jeweilige Impfstoffcharge darf in Deutschland nur dann in den Verkehr gebracht werden, wenn das PEI als offizielle Arzneimitteluntersuchungsstelle diese freigegeben hat. Die Chargenprüfung beinhaltet neben der experimentellen Prüfung der Impfstoffe auch die Überprüfung der chargenspezifischen Daten zur Herstellung und Qualitätskontrolle, die der Hersteller mit dem Antrag auf Chargenfreigabe einzureichen hat. Als OMCL führt das PEI die in den europäischen impfstoffspezifischen Leitlinien zur Chargenprüfung (Batch Release Guidelines) festgelegten experimentellen Prüfungen unabhängig vom Hersteller durch, um die zulassungskonforme Qualität jeder Impfstoffcharge unabhängig zu bestätigen [4, 11].

#### OCABR-Verfahren und -Leitlinien

Wenn ein OMCL auf nationaler Ebene das OCABR-Verfahren implementiert, folgt es dem zentralen Verwaltungsverfahren für humanbiologische Arzneimittel, dem „EU Administrative Procedure for Official Control Authority Batch Release“. Dieses öffentlich zugängliche Dokument ist auch für die Verwendung durch den Zulassungsinhaber vorgesehen und dient als Leitfaden für die Kommunikation zwischen Zulassungsinhaber und OMCL [8].

Das EU-Verwaltungsverfahren für die Chargenfreigabe durch die amtlichen Kontrollbehörden definiert den rechtlichen Rahmen, den Zweck, die Grundsätze, das Verfahren für OCABR bzw. den OCABR-Prozess und skizziert die Schritte, die von OCABR-beteiligten Mitgliedstaaten und Antragstellern zu unternehmen sind. Arzneimittelhersteller mit OCABR-pflichtigen Arzneimitteln sollten so früh wie möglich Kontakt mit einem OMCL aufnehmen. Wie in den Verfahrenshinweisen der Europäischen Arzneimittel-Agentur (European Medicines Agency, EMA) für Anwender eines zentralisierten Verfahrens erwähnt, wird Antragstellern dringend empfohlen, frühzeitig eine Zusammenarbeit mit einem OMCL einzugehen. Diese Zusammenarbeit sollte früh genug begonnen werden, um die Implementierung der angeforderten Methoden (definiert in der OCABR-Richtlinie) rechtzeitig für die erste Chargenfreigabe zu ermöglichen. Weiterhin wird empfohlen, dass der Antragsteller ein zweites OMCL für die Chargenfreigabe des zugelassenen Produkts benennt, um die Lieferkette zu sichern und das Risiko von Lieferengpässen aufgrund von fehlenden Testkapazitäten zu minimieren. Um ein offizielles Chargenfreigabezertifikat der zuständigen Kontrollbehörde zu erhalten, reicht der Zulassungsinhaber Proben der freizugebenden Charge zusammen mit Produktions- und Kontrollprotokollen bei einem OMCL ein.

Zusätzlich zum EU-Verwaltungsverfahren gibt es eine Reihe produktspezifischer Leitlinien für Humanimpfstoffe. Diese werden von einem Expertengremium aus verschiedenen OMCL und mit Unterstützung der EDQM ausgearbeitet und berücksichtigen den aktuellen Stand der Methodik und Techniken z. B. für Impfstoffe, wie sie in den Zulassungen beschrieben sind. Die Leitlinien werden vom OMCL-Netzwerk erst nach Konsultation innerhalb des Netzwerks und gegebenenfalls nach einer öffentlichen Befragung unter Beteiligung interessierter Kreise angenommen und gewährleisten einen gemeinsamen Testansatz in allen OMCL und Transparenz für alle Benutzer des Systems. Die produktspezifischen Leitlinien beinhalten eine Auflistung der Tests, die vom OMCL für die Chargenfreigabe der jeweiligen Arzneimittel durchgeführt werden sollen. Die festgelegten Tests wurden vorher, einschließlich der für die durch die EMA zentralisiert zugelassenen Produkte, vom OCABR-Netzwerk aus den vom Hersteller gemäß dem Zulassungsdossier für das Produkt durchgeführten Tests ausgewählt und vereinbart. In den Leitlinien stehen den Antragstellern auch Mustervorlagen für die Chargenfreigabe-Protokolle zur Verfügung, um die Antragstellung zu erleichtern, einen harmonisierten Ansatz zu fördern und sicherzustellen, dass alle relevanten Informationen enthalten sind. Sowohl das Dokument zum EU-Verwaltungsverfahren als auch die europäischen produktspezifischen Leitlinien sind auf der Internetseite der EDQM verfügbar [6, 8, 12].

Das Chargenfreigabeverfahren der offiziellen Kontrollbehörden innerhalb der EU besteht also aus einer kritischen Bewertung des Produktions- und Kontrollprotokolls des Herstellers und der Prüfung von Proben, die vom Hersteller gemäß den produktspezifischen Leitlinien eingereicht wurden. Darüber hinaus ist es wichtig zu beachten, dass die Chargenfreigabe der offiziellen Kontrollbehörden auf Grundlage eines etablierten Qualitätsmanagementsystems durchgeführt wird, das einer regelmäßigen externen Bewertung auf der Grundlage des internationalen Standards ISO 17025 unterzogen wird [6, 8].

Um den Freigabeprozess von Impfstoffchargen zu beschleunigen, werden die experimentellen Prüfungen in den OMCL in den meisten Fällen zeitgleich mit den Prüfungen beim Hersteller durchgeführt. Dieses Vorgehen wird als Parallel-Testung (Parallel Testing) bezeichnet [8, 11].

#### Nichtkonformität sowie Unterbrechung und Rücknahme während des Parallel-Testungsverfahrens

Sind alle Kriterien für die Chargenfreigabe erfüllt, stellt das zuständige OMCL ein OCABR-Zertifikat aus. Wird jedoch festgestellt, dass eine Charge nicht den Spezifikationen entspricht, werden diese Informationen dem Zulassungsinhaber und bestimmten Kontaktpersonen im EU-OCABR-Netzwerk (einschließlich OMCL, zuständige Behörden, EMA, EU-Kommission, EDQM, Department of Biological Standardisation, OMCL-Networks and HealthCare Department (DBO) und allen OCABR-Netzwerkmitgliedern mit Beobachterstatus, die durch ein spezifisches Netzwerkverfahren als Beobachter zugelassen sind) zur Verfügung gestellt. Weiterhin erhält die Charge bei einem ungenügenden Prüfungsergebnis eine Bescheinigung über die Nichtkonformität und darf nicht in Verkehr gebracht werden. Alle Mitglieder des OCABR-Netzwerks werden über die fehlende Konformität der betroffenen Charge informiert.

Bei der Parallel-Testung fallen alle Chargen, welche die Tests nicht bestehen und anschließend vom Hersteller vor Abschluss des OCABR-Verfahrens zurückgezogen werden, formal nicht unter den Begriff der Nichtkonformität. Informationen über die Rücknahme werden dennoch innerhalb des OCABR-Netzwerks verbreitet, um so zu vermeiden, dass diese Chargen erneut bei einem anderen OMCL eingereicht werden könnten [6, 8].

## Vergangene und zukünftige Herausforderungen am Beispiel von COVID-19-Impfstoffen: ein OMCL-Praxisbericht

### Von der Implementierung bis zur ersten Chargenfreigabe

Sobald die Hersteller von COVID-19-Impfstoffen für die Zulassung bereit waren, begannen sie, nach geeigneten OMCL für die experimentellen Prüfungen für die Chargenfreigabe zu suchen. Um die Suche nach OMCL zu erleichtern, koordinierte die EDQM die Kapazitäten/Methoden, die bei jedem OMCL verfügbar sind. Das PEI wurde für die Prüfung und Freigabe von mRNA- und Adenovirus-basierten Impfstoffen ausgewählt. Danach wurde mit dem Transfer der Chargenfreigabemethoden begonnen. Diese Tests waren bereits im Vorfeld für die Erstellung der europäischen produktspezifischen Leitlinien ausgewählt und festgelegt worden. Alle erforderlichen Materialien und Methoden wurden vom Hersteller und OMCL gemeinsam genutzt, um die zeitnahe Etablierung und Validierung jeder Methode zu ermöglichen.

Grundsätzlich umfassen die vom OMCL durchzuführenden experimentellen Chargenprüfungen folgende Qualitätsindikatoren der Impfstoffchargen: visuelle Überprüfung der Beschaffenheit, Nachweis der Identität der im Impfstoff enthaltenen wirksamen Substanz, Wirkstoffgehalt (Potency) sowie Integrität der wirksamen Substanz.

Um Lieferengpässe bei Impfstoffen zu vermeiden, erfolgt häufig eine Parallel-Testung bei den Impfstoffherstellern und den OMCL. Konkret bedeutet dies, dass bei der Produktion einer Charge ein Teil der abgefüllten Behälter an die Qualitätskontrollabteilung des Herstellers sowie an das OMCL geschickt wird. Dabei muss der Impfstoffhersteller eine deutlich umfangreichere Liste von experimentellen Prüfungen als das OMCL durchführen, die im Rahmen der Zulassung festgelegt worden sind. Dagegen müssen die OMCL für die Chargenprüfung nur die Tests wiederholen, die speziell in den europäischen produktspezifischen Leitlinien definiert sind. Die für die COVID-19-Impfstoffe vorhandenen Leitlinien sind auf der Internetseite der EDQM verfügbar [12]. Des Weiteren sind die von den OMCL durchzuführenden Chargenfreigabetests für die COVID-19-Impfstoffe in der Tab. 2 zusammengefasst [13–16]. Aufgrund der Parallel-Testung sind die Tests beim OMCL bereits abgeschlossen, sobald die Dokumentation des Herstellers für die offizielle Chargenfreigabe fertiggestellt ist. Daher gibt es auf OMCL-Ebene keine Verzögerung für die Chargenfreigabe. Wenn der Impfstoffhersteller die Chargenfreigabe für eine Charge beantragt, überprüft das zuständige OMCL alle bereitgestellten Unterlagen sowie die von beiden Seiten erzielten Ergebnisse und gibt die Charge frei, sollte die Bewertung zufriedenstellend sein.

**Table 2:** 

	COVID-19-Impfstoff (nicht-replizierender Adenovirus-Vektorimpfstoff)	COVID-19-mRNA-Impfstoff	COVID-19-Impfstoff (rekombinanter Proteinimpfstoff)	COVID-19-Impfstoff (inaktiviert)
Testdurchführung an der finalen Charge (Final Lot)	Visuelle Kontrolle	Visuelle Kontrolle	Visuelle Kontrolle	Visuelle Kontrolle
	Wirkstoffgehalt	Wirksamkeit	Wirksamkeit	Wirksamkeit
	Identität	Identität	Identität	Identität
	–	Integrität	Reinheit	–
Testdurchführung an Vorstufe der finalen Charge (Final Bulk)	–	–	Reinheit (wenn nicht an finaler Charge durchgeführt)	–

Die zeitgleiche Durchführung der experimentellen Prüfungen beim Hersteller und beim OMCL ist ein allgemeines Verfahren, das den Herstellern von allen OMCL empfohlen wird. Im Falle der COVID-19-Impfstoffe war der Austausch von Methodeninformationen mit dem Fortschritt der Zulassung verknüpft. Die vom PEI durchzuführenden Chargenfreigabetests wurden vor der offiziellen Erteilung der Zulassung für die Impfstoffe etabliert und validiert. Dadurch war sichergestellt, dass die Chargenprüfung am PEI unverzüglich durchgeführt werden konnte und die Chargenprüfung durch das OMCL zu keinerlei Verzögerung der Marktverfügbarkeit der Impfstoffchargen führte. Angesichts der hohen Nachfrage nach COVID-19-Impfstoffen wurden die experimentellen Prüfungen vorrangig durchgeführt, sobald eine Impfstoffcharge eingereicht wurde, und freigegeben, sobald die Überprüfung und Bewertung der Dokumente und Daten sowie die Testergebnisse zufriedenstellend waren.

### Beitrag zur europäischen und globalen Versorgung mit COVID‑19-Impfstoffen

Obwohl das Prinzip der Chargenprüfung/Chargenfreigabe für alle Humanimpfstoffe ähnlich ist, war bei den COVID-19-Impfstoffen neben der Entwicklung und Herstellung eine der großen Herausforderungen zur Gewährleistung einer ausreichenden Marktversorgung die Sicherstellung der zeitnahen unverzüglichen Chargenprüfung. Insbesondere in der frühen Phase nach der Zulassung der ersten Impfstoffe und der hohen Erwartung der Bevölkerung, zeitnah Zugang zu diesen Impfstoffen zu bekommen, war es von größter Wichtigkeit sicherzustellen, dass es an dieser Stelle zu keiner Unterbrechung des Versorgungsprozesses kommt. Im PEI wurde ein Schichtbetrieb eingeführt, um die experimentelle Prüfung aller Chargen für die Belieferung des europäischen und des deutschen Marktes konstant fortzusetzen und um zu vermeiden, dass im Falle von SARS-CoV-2-Infektionen einzelner Mitarbeitender die komplette Chargenprüfung wegen notwendiger umfangreicher Quarantänemaßnahmen zum Erliegen käme. Weiterhin war es eine Herausforderung, die regelmäßige Chargenfreigabe für alle nichtpandemischen Impfstoffe (wie z. B. Masern-Mumps-Röteln- oder Influenzaimpfstoffe) fortzusetzen und daneben die neuen Methoden für die COVID-19-Impfstoffe in Wechselschichten zu etablieren und zu validieren. Dank dieser und anderer organisatorischer Maßnahmen konnte sichergestellt werden, dass es seitens des PEI zu keiner Verzögerung bei der Chargenfreigabe für einen der getesteten Impfstoffe kam.

Das PEI gab als erstes OMCL am 23.12.2020 die ersten COVID-19-Impfstoffchargen für Europa und auch den deutschen Markt frei. Somit konnten alle von den OMCL freigegebenen COVID-19-Impfstoffchargen unmittelbar EU-weit zur Verfügung gestellt werden. Zusätzlich zur Versorgung des europäischen Marktes koordinierte die weltweite Initiative COVAX internationale Ressourcen, um Ländern mit niedrigem bis mittlerem Einkommen einen gerechten Zugang zu COVID-19-Tests, -Therapien und -Impfstoffen zu ermöglichen. Das PEI führte zusätzlich zur Ausstellung von EU/EAA-OCABR-Zertifikaten und nationalen Freigabebescheiden auch Prüfungen für die WHO-Zertifizierung durch. Dies ermöglichte aufgrund der durchgeführten OCABR-Testungen die Freigabe der COVID-19-Impfstoffe auch in Ländern außerhalb Europas. Hier erfolgte die erste WHO-Freigabe im Februar 2021.

Es ist wichtig zu beachten, dass der Bedarf an COVID-19-Impfstoffen seit Dezember 2020 nicht zurückgegangen ist. Dies ist durch die Empfehlungen für Auffrischimpfungen, aber auch durch die mittlerweile erfolgte Genehmigung von an SARS-CoV-2-Varianten adaptierten Impfstoffen begründet. Auch wenn zukünftig die Nachfrage in Europa abnehmen sollte, so ist die weltweite Versorgung mit COVID-19-Impfstoffen, die zur Beendigung der Pandemie äußerst wichtig ist, noch nicht abgeschlossen.

### Zukünftige Virusvarianten: Stammanpassung

Die ständige Veränderung von SARS-CoV‑2 führt zu einer immer stärker werdenden Immunevasion (Immune Escape) von der durch die Impfung induzierten Immunantwort. Insbesondere durch die seit November 2021 zirkulierende Omikron-Variante ist die Wirksamkeit der COVID-19-Impfung zunehmend vermindert [17]. Grundsätzlich ist dieses Problem auch bei anderen Erregern bekannt, insbesondere bei den Influenzaviren. Daher ist die Anpassung der Impfstoffe an neue besorgniserregende Varianten von enormer Bedeutung [18, 19].

Da von einer parallelen Existenz verschiedener Virusvarianten (ggf. auch mit unterschiedlicher geografischer Verbreitung) auszugehen ist, kann eine Strategie die Entwicklung von bivalenten COVID-19-Impfstoffen sein. Im Falle der derzeit zugelassenen Impfstoffe würde dies bedeuten, dass sie dann auf den Spikeproteinen zweier unterschiedlicher Varianten basieren. Dabei ist zu berücksichtigen, dass mit steigender Menge an mRNA die Reaktogenität der Impfstoffe zunimmt. Daher wird bei bivalenten Impfstoffen die Gesamtmenge an mRNA gegenüber dem monovalenten Impfstoff nicht erhöht, sondern verteilt sich 1:1 auf die beiden Komponenten. Seit September 2022 sind auf der Basis einer Zulassungsvariation auch folgende bivalente Impfstoffe in der EU verfügbar: Comirnaty Original/Omicron BA.1 (BioNTech/Pfizer), bei dem 15 µg mRNA für das Spikeprotein des Ursprungsisolats codiert und 15 µg mRNA für das Spikeprotein der Omikron-Variante BA.1, bzw. Spikevax bivalent Original/Omicron BA.1 (Moderna) mit 25 µg mRNA, die für das Spikeprotein des Ursprungsisolats codiert und 25 µg mRNA für das Spikeprotein der Omikron-Variante BA.1. Der an die seit Mitte des Jahres 2022 in Europa vorherrschende Omikron-Variante BA.5 angepasste Impfstoff Comirnaty Original/Omicron BA.4–5 enthält 15 µg mRNA, die für das Spikeprotein des Ursprungsisolats codiert und 15 µg mRNA für das Spikeprotein der Omikron-Varianten BA.4 und 5. Dies wiederum bedingt neue Herausforderungen hinsichtlich der durchzuführenden Tests.

## Fazit

Am Beispiel der COVID-19-Impfstoffe konnte gezeigt werden, dass die Chargenprüfung eine wesentliche Säule der Bereitstellung sicherer und wirksamer Impfstoffe ist.

Die Überwachung von Impfstoffen durch OMCL wie das PEI trägt dazu bei, den nationalen und internationalen Markt mit sicheren, wirksamen und qualitativ hochwertigen Impfstoffen zu versorgen. Auch bei den COVID-19-Impfstoffen wurden die Chargenprüfungen und -freigaben gemäß dem OCABR-Verfahren und allen gesetzlichen Anforderungen durchgeführt, jedoch mit kürzeren Fristen. Dies stellte eine große Herausforderung dar, deren Bewältigung sich aber für die Sicherheit der Bevölkerung und Bewältigung der Pandemie lohnt.
